# Dynamic and Physical Clustering of Gene Expression during Epidermal Barrier Formation in Differentiating Keratinocytes

**DOI:** 10.1371/journal.pone.0007651

**Published:** 2009-10-30

**Authors:** Jennifer M. Taylor, Teresa L. Street, Lizhong Hao, Richard Copley, Martin S. Taylor, Patrick J. Hayden, Gina Stolper, Richard Mott, Jotun Hein, Miriam F. Moffatt, William O. C. M. Cookson

**Affiliations:** 1 Wellcome Trust Centre for Human Genetics, Oxford, England; 2 Oxford Centre for Gene Function, Department of Statistics, University of Oxford, Oxford, England; 3 National Heart and Lung Institute, Imperial College, London, England; 4 MatTek Corporation, Ashland, Massachusetts, United States of America; Centre for Genomic Regulation, Spain

## Abstract

The mammalian epidermis is a continually renewing structure that provides the interface between the organism and an innately hostile environment. The keratinocyte is its principal cell. Keratinocyte proteins form a physical epithelial barrier, protect against microbial damage, and prepare immune responses to danger. Epithelial immunity is disordered in many common diseases and disordered epithelial differentiation underlies many cancers. In order to identify the genes that mediate epithelial development we used a tissue model of the skin derived from primary human keratinocytes. We measured global gene expression in triplicate at five times over the ten days that the keratinocytes took to fully differentiate. We identified 1282 gene transcripts that significantly changed during differentiation (false discovery rate <0.01%). We robustly grouped these transcripts by K-means clustering into modules with distinct temporal expression patterns, shared regulatory motifs, and biological functions. We found a striking cluster of late expressed genes that form the structural and innate immune defences of the epithelial barrier. Gene Ontology analyses showed that undifferentiated keratinocytes were characterised by genes for motility and the adaptive immune response. We systematically identified calcium-binding genes, which may operate with the epidermal calcium gradient to control keratinocyte division during skin repair. The results provide multiple novel insights into keratinocyte biology, in particular providing a comprehensive list of known and previously unrecognised major components of the epidermal barrier. The findings provide a reference for subsequent understanding of how the barrier functions in health and disease.

## Introduction

**Figure 1:**
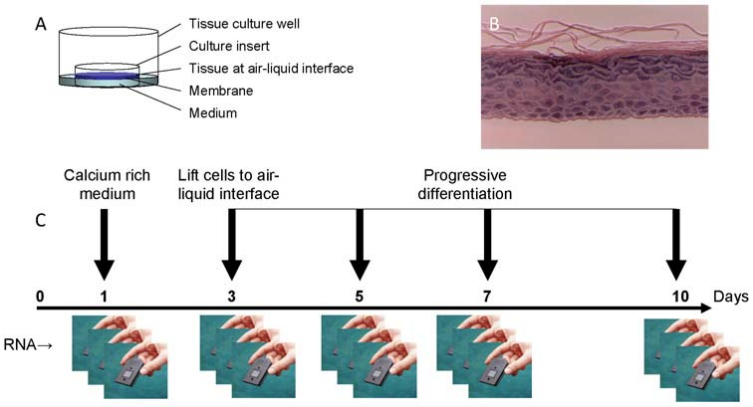
The EpiDerm™ model of keratinocyte differentiation. The figure shows a schematic of the experimental design including the culture arrangements (A). The fully differentiated model (B) (MatTek Corporation) contains organized basal, spinous, granular, and cornified layers analogous to those found in normal human skin. The course of the experiment (C) includes biological triplicates taken for microarray analyses at five points, starting before the addition of calcium to the culture medium.

Keratinocytes develop continuously from a basal layer replenished by stem cells in hair follicles. Primary keratinocytes can be induced to form a differentiated model of the epidermis by the addition of calcium and culture at an air-liquid interface on membrane inserts. In our study, we have investigated a commercial human keratinocyte model (EpiDerm™, MatTek Co) that uses serum free medium and exhibits uniform and reproducible growth to obtain organized basal, spinous, granular, and cornified layers analogous to those found in normal human skin (Figure 1).

We harvested cells from the model at five times, starting before calcium was added to the medium (0 days), immediately before the cultures were lifted to the air-liquid interface (3 days) and three subsequent time points at five, seven and ten days after calcium addition (Figure 1). Each time was tested in triplicate from different cultures.

We analysed this time-series gene expression data to identify the transcripts that varied most significantly during differentiation, and we then used a clustering algorithm to find genes with correlated expression patters. We tested genes within each expression cluster for shared regulatory motifs. In order to understand the biological significance of our results, we examined the gene ontology (GO) content of each cluster and used known genes to suggest the function of other cluster members.

We also carried out detailed examination of transcripts from the key genomic regions of the MHC and the Epidermal Differentiation Complex (EDC). We in addition examined genes with specific GO descriptors such as transcription factors or immune response.

## Results

### Expression Clusters (exCs)

RNA was extracted, converted to cRNA and hybridised to Affymetrix HG-U133A GeneChip® arrays (data deposited in MIAMEXPRESS with accession number E-MEXP-1130). The arrays contained a total of 22,283 probe set elements, representing 11,870 unique Ensembl gene identifiers. We pre-processed and normalised raw signal data according to Affymetrix recommendations [1]. We only included those probe sets with signal robustly greater than background in all three replicates at any one time for further analysis. Multiple probe sets from the same gene were grouped into a single datum when the expression was correlated (*r*>0.75). We identified a group of 1,282 probe sets showing statistically significant differences in levels of expression between the five times with the Significance Analysis of Microarrays (SAM) method [2], using a stringent threshold false discovery rate (FDR) <0.01%.

**Figure 2:**
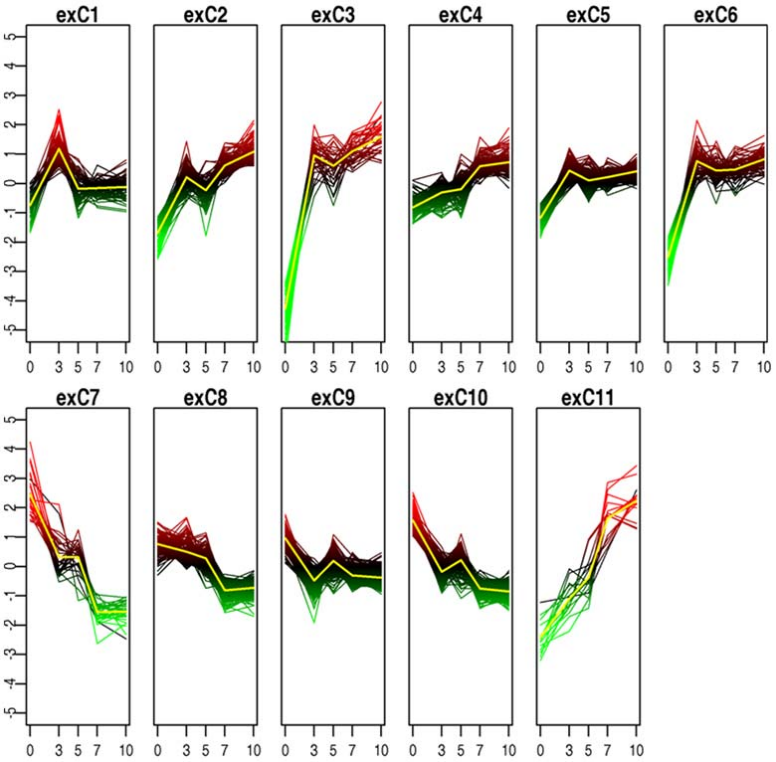
K-means clustering of genes differentially expressed during keratinocyte differentiation. The mean abundance of each transcript at each of the 5 time points is shown for each of the 11 clusters of gene expression. Abundance levels are log base 2 with mean 0.

We used a robust modification of K-means clustering [3] to group these 1,282 transcripts into 11 discrete and stable clusters (Figure 2) (Table S1). Clusters retained a high proportion (mean = 92.29%±7.2 SD) of members across 100 clustering outcomes, each randomly seeded 1000 times.

The clusters showed distinctive temporal patterns characterised by their initial levels of abundance and variation during differentiation (Figure 2). Expression clusters exC1-6 and exC11 showed a general increase in abundance across the time course, whereas members of exC7-10 showed a general decrease. ExC3, exC6 and exC11 could be separated from other clusters showing increases of abundance because of marked (over 60 fold) increases in gene expression levels. Members of exC11 showed continued increases in expression levels over the entire process of differentiation.

### Regulatory Elements

We investigated whether these clusters were attributable to biological co-regulation by seeking common *cis*-regulatory elements within individual exCs. We used the Weeder algorithm^11^ to identify the 50 highest scoring ‘sequence words’ between −2 kb and +0.5 kb of transcription start sites relative to a background set of approximately 26,000 human promoter sequences (Table S2). We found that across exCs an average of 89.6%±20.6 SD of the highest scoring words reached significance (*P*≤0.05), and 95.4%±8.4 SD of the highest scoring words were significantly enriched in any one exC relative to the permuted dataset. This indicated a high-degree of exC-specific regulatory element annotation.

**Figure 3:**
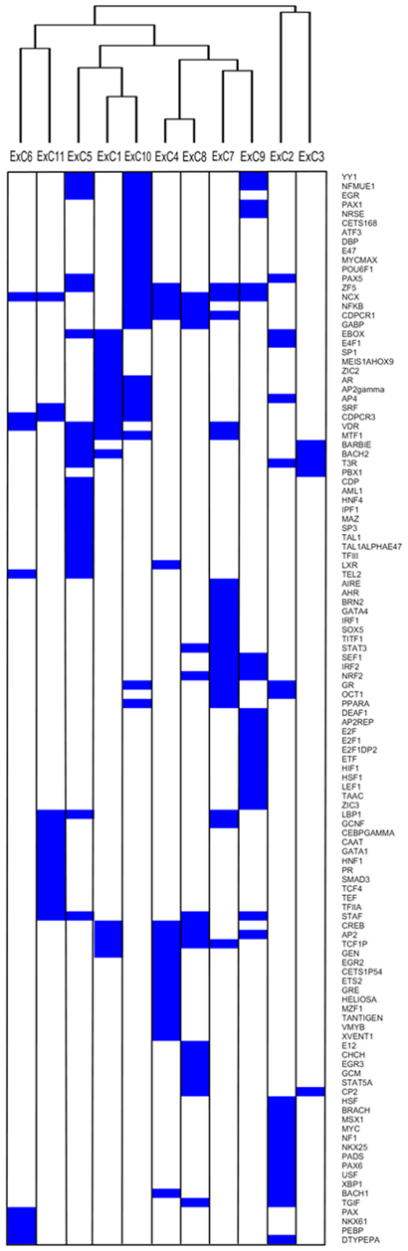
Cluster analysis of putative transcription factor binding sites for each expression cluster. The figure shows potential binding site motifs for *cis*-acting regulatory factors in sequences 2 Kb upstream and 0.5 Kb downstream of transcripts in each expression cluster. Blue boxes indicate the significant presence of a putative binding site, with the TRANSFAC identifier to the right of the figure. Binding sites (rows) and expression clusters (columns) are ordered based on hierarchical clustering using a binary distance metric and complete linkage. The dendrogram at the top displays the distance between expression clusters as calculated from comparison of binding site profiles.

We visualised regulatory themes within exCs using hierarchical clustering of the presence of significantly scoring binding site motifs, further indicating the presence of exCs-specific patterns (Figure 3). These results suggest that, at least to some extent, the expression clusters represent biologically co-regulated genes.

### Gene Ontology and Function within Expression Clusters

We found distinctive functional correlates for the various exCs (Table 1 and Table S3), through extraction of Gene Ontology (GO) [4] terms for the Affymetrix HG-U133A chip from Ensembl.

**Table 1:** Summary of Gene Ontology of expression clusters in differentiating keratinocytes.

	BIOLOGICAL PROCESS			MOLECULAR FUNCTION		
Cluster	Name	LOR^a^	*P_c_* b	Activity	LOR^a^	*P_c_* b
**3**	Ectoderm development	1.765	≤1e-7	Protease inhibitor	1.401	2e-4
	Epidermis development	1.86	≤1e-7	Endopeptidase inhibitor	1.403	2e-4
	Histogenesis	1.462	2e-7			
	Keratinization	2.362	9e-5			
**4**				Oxidoreductase	1.093	1e-4
**6**	Epidermis development	1.496	1e-7	Carbonate dehydratase	1.769	2e-4
	Ectoderm development	1.401	9e-7	Hydro-lyase	1.367	0.006
	Histogenesis	1.156	1e-5	Carbon-oxygen lyase	1.321	0.009
	Keratinization	2.036	0.002			
	One-carbon compound metabolism	1.418	0.006			
**7**	Cell motility	1.372	2e-5	Heparin binding	1.826	≤1e-7
	Epidermis development	1.72	2e-5	Glycosaminoglycan binding	1.693	≤1e-7
	Ectoderm development	1.625	5e-5	Polysaccharide binding	1.687	≤1e-7
	Cell adhesion	1.068	1e-4	Protease inhibitor	1.394	0.003
	Blood coagulation	1.468	0.003			
	Wound healing	1.455	0.004			
**8**	Carbohydrate catabolism	1.236	≤1e-7	Oxidoreductase	1.527	1e-6
	Alcohol catabolism	1.272	≤1e-7	Growth factor	0.846	0.003
	Blood coagulation	0.983	0.007			
	Wound healing	0.97	0.008			
**9**	RNA processing	0.694	≤1e-7	Unfolded protein binding	0.891	≤1e-7
	Primary metabolism	0.391	≤1e-7	RNA binding	0.598	≤1e-7
	Protein folding	0.823	≤1e-7	ATP binding	0.383	2e-4
	DNA replication	0.914	<1e-7	Adenyl nucleotide binding	0.374	4e-4
**10**	Mitotic cell cycle	0.966	≤1e-7	Cadmium ion binding	1.794	0.007
	Cell cycle	0.718	≤1e-7			
	Cell division	1.069	≤1e-7			
**11**	Digestion	2.057	1e-5	Aldo-keto reductase	2.665	5e-6
	Lipid metabolism	1.163	0.006	Oxidoreductase	2.457	2e-5
	Monocarboxylic acid transport	2.156	0.009	Bile acid transporter	2.53	0.001

a LOR = log odds ratio of GO term relative to all other probes on the H133A chip. Results for GO terms for cellular localisation and negative associations are given in Table S2.

b *P_c_* = corrected *P* values.

ExC3 and exC6 showed the greatest increases in gene expression levels during the experimental time course (Figure 2). Their distinctive pattern of high late expression was consistent with elements that construct the skin barrier and other defences of the epithelium. Consistent with this hypothesis, the exC3 cluster contained two genes (*SPINK5* and *FLG*) previously recognised to confer susceptibility to childhood atopic dermatitis (AD) [5], [6], and one (*CDSN*) which has been implicated in psoriasis[7], [8]. In addition, exC3 and exC6 were significantly enriched (*P*<10^−7^) for genes with GO descriptors for epidermis development (Table 1).

We therefore examined exC3 for other genes which may contribute to the defences of the epidermis. The cluster contained a superabundance of protease inhibitors (*P* = 0.0002) (Table 1 and Table 2). These included *SPINK5*, which is a polyvalent protease inhibitor, and four other protease inhibitors (Cystatin 6 (*CST6*), *SERPINB3*, *SERPINB4* and *PI3* (Elafin, *SKALP*)). *PI3* is an endogenous inhibitor of neutrophil elastase with intrinsic antimicrobial activity [9], [10], and *SERPINB4* inhibits the major dermatolytic *Der P* I protease from the house dust mite [11]. These results suggest by implication a novel role for *SERPINB3* and *CST6* in barrier function and innate immunity.

**Table 2:** Genes forming the epithelial barrier (expression cluster 3).

Gene	Symbol	Location
Calmodulin-like 5	CALML5	10p15.1
Cystatin E/M	CST6	11q13
Aldehyde dehydrogenase 3 family, member B2	ALDH3B2	11q13
Keratin 1 (epidermolytic hyperkeratosis)	KRT1	12q12-q13
Carboxypeptidase M	CPM	12q14.3
Histidine ammonia-lyase	HAL	12q22-q24.1
Protein kinase H11	H11	12q24.23
RAR-related orphan receptor A	RORA	15q21-q22
V-maf musculoaponeurotic fibrosarcoma oncogene homolog	MAF	16q22-q23
Arachidonate 12-lipoxygenase, 12R type	ALOX12B	17p13.1
Keratin 10	KRT10	17q21
Keratin 23	KRT23	17q21.2
Desmoglein 1	DSG1	18q12.1
Desmocollin 1	DSC1	18q12.2
Serine (or cysteine) proteinase inhibitor, clade B, member 3	SERPINB3	18q21.3
Serine (or cysteine) proteinase inhibitor, clade B, member 4	SERPINB4	18q21.3
Kallikrein 7 (chymotryptic, stratum corneum)	KLK7	19q13.41
S100 calcium binding protein A8 (calgranulin A)	S100A8	1q21
Small proline rich-like (epidermal differentiation complex) 1B	SPRL1B	1q21
NICE-1 protein	NICE-1	1q21
S100 calcium binding protein A9 (calgranulin B)	S100A9	1q21
Filaggrin	FLG	1q21
Loricrin	LOR	1q21
Small proline-rich protein 3	SPRR3	1q21-q22
Small proline-rich protein 2B	SPRR2B	1q21-q22
Protease inhibitor 3, skin-derived (SKALP)	PI3	20q12-q13
Hypothetical protein FLJ10134	FLJ10134	3q12.3
S100 calcium binding protein P	S100P	4p16
Homeodomain-only protein	HOP	4q11-q12
Hydroxyprostaglandin dehydrogenase 15-(NAD)	HPGD	4q34-q35
Serine protease inhibitor, Kazal type, 5	SPINK5	5q32
Corneodesmosin	CDSN	6p21.3
Lymphocyte antigen 6 complex, locus G6C	LY6G6C	6p21.31
Elongation of very long chain fatty acids like 4	ELOVL4	6q14
Arginase, liver	ARG1	6q23
Early growth response 3	EGR3	8p23-p21
Chloride intracellular channel 3	CLIC3	9q34.3

Other genes within exC3 encoded structural proteins such as keratins 1, 10 and 23, desmogelin 1 (*DSG1*), desmocollin 1 (*DSC1*) and CDSN. These proteins may be modified by proteases such as Kallekrein 7 *(KLK7)* and carboxipeptidase M *(CPM)* from the same cluster to regulate growth and desquamation [12], [13].

Several genes from exC3, including the *S100* and *SPRR* genes, *CST6* and *SERPINB3* are used as indirect markers of epithelial differentiation in malignancy, where loss of differentiation is associated with poor outcomes. The *MAF* oncogene, *HOP*, *RORA* and *EGR3* from the same cluster directly participate in the regulation of growth, and as a consequence may be better markers of cancer differentiation status.

Amongst the other exCs with increasing abundance, genes in exC4 predominately mediated fatty acid metabolism (*P* = 0.0001), genes in exC6 displayed lyase activity (*P* = 0.006) and genes in exC11 were principally involved in lipid metabolism (*P* = 0.00001). The functions of exC11 genes may reflect the integration of lipids into the insoluble outer cornified envelope of the epidermis as differentiation progresses.

ExC7 (Figure 2, Table S1), which showed a steep and continued decline in abundance from an initial high, was enriched for genes involved in cell motility and development (*P* = 0.00002), cell adhesion (*P* = 0.0001) and blood coagulation (*P* = 0.003). It is possible that the genes in this cluster define a phenotype for motile undifferentiated keratinocytes. This motility is likely to be important for re-epithelialisation during wound healing. Genes with immune-related functions also declined as the keratinocytes developed (Table S5 and discussed below in our findings for the MHC), suggesting that immunological activity is also a characteristic of undifferentiated keratinocytes.

ExC8, which was typified by a more gradual decline in expression levels than exC7, was also characterised by genes for blood coagulation (*P* = 0.007) and wound healing (*P* = 0.008). In addition, it contained many genes involved in glucose metabolism, (*P*<10^−7^) perhaps reflecting declining energy usage of differentiating cells.

ExC9 showed an initial decline followed by a rise and a second fall and contained many genes for RNA processing, protein folding and DNA replication (*P*<10^−7^ for each category) (Table 1), reflecting dynamic transcriptional and translational activity of keratinocytes throughout differentiation. ExC10 showed a similar but more pronounced pattern of decline and contained an excess of genes with GO items for mitosis, cell cycle and cell division (*P*<10^−7^ for each category), reflecting the decreasing activity of these functions as the cell model terminally differentiated.

### Physical Clustering

**Figure 4:**
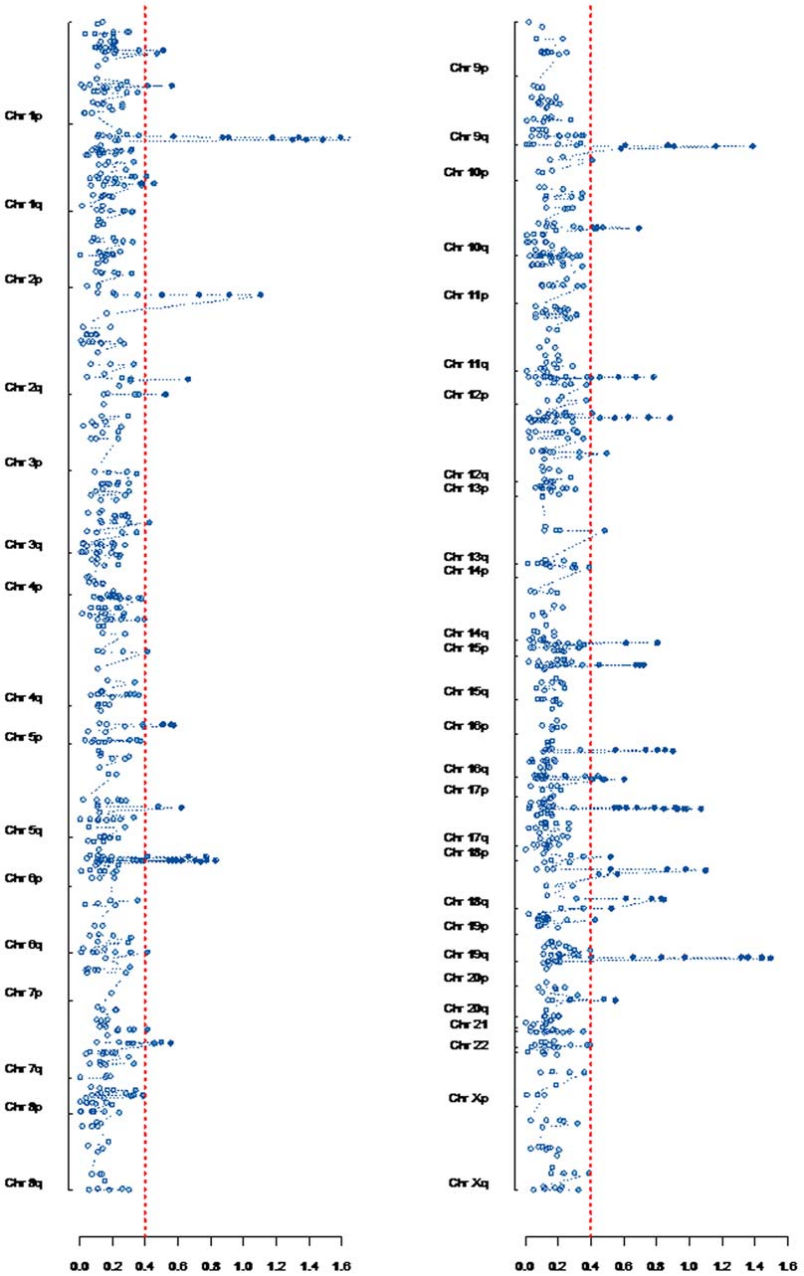
Physical clustering of genes differentially expressed during keratinocyte differentiation. The human genome is depicted in two vertical panels with p and q arms labelled. All probe sets detected as differentially abundant across the time course are displayed at their mapped physical distance within each chromosome and their corresponding Smith-Waterman (SW) score (x axis). The significance threshold corresponding to a FDR of 1 false positive is shown in red. The maximum SW score of 4.1 was detected within the epidermal differentiation complex (EDC) on chromosome 1p21.

Physical clustering of genes in discrete chromosomal locations is a potential mechanism for effecting co-regulation of expression., although it has been assumed to be of minor importance in the human genome [14]. We therefore sought for possible physical clusters (pCs) among the 1,282 transcripts that varied in abundance during differentiation by using a modified Smith-Waterman algorithm [15]. After correction for multiple testing, we observed 29 statistically significant pCs (designated pC-KD1 - 29) within the above list (Figure 4) (Table S4). This suggested that physical clustering is a frequent mechanism for gene co-regulation in differentiating keratinocytes.

We observed an incomplete correlation between physical and expression clusters. Genes within individual pCs often showed different abundance profiles across the time course and were expressed in both directions from complementary strands of DNA (Table S4). We exemplified these observations in the detailed analysis of the Epidermal Differentiation Complex (EDC, containing pC-KD4) and the MHC (containing pC-KD12 and pC-KD13) described below.

Of the 29 pCs detected, 4 consisted entirely of paralogs such as the keratin genes in pC-KD16 and pC-KD23 on chromosomes 12q12-13 and 17q21, and the kallikrein genes in pC-KD28 on chromosome 19q13. These results are dependent on the ability of the Affymetrix probe designs to discriminate between related sequences. Sequence alignment of the probes for these genes suggested cross-hybridisation within these families was unlikely. Eleven pCs contained mixtures of paralogs and other genes, so that 15 (52%) of all pCs might be considered to have arisen from gene duplications (Table S4). The remaining pCs contained genes with diverse sequences and functions (Table S4).

The results suggest gene duplication is an important factor in developing pCs, but that other mechanisms play a significant part.

We found that expression profiles were not uniform within gene families. *KRT1*, *KRT2* and *KRT10* are, for example, commonly used as markers of keratinocyte differentiation [16]. Our results found that *KRT4*, *KRT16* and *KRT23* also progressively increased in abundance. By contrast *KRT7*, *KRT8*, *KRT15* and *KRT19* showed neutral or declining transcript levels (Figure S1), even though they shared the same pCs as genes with increasing abundance.

Previous studies of Serial Analysis of Gene Expression (SAGE) in multiple tissues have shown significant physical clustering that was attributable to housekeeping genes rather than expression of tissue-specific transcripts [17]. The pCs we observed do not contain an abundance of housekeeping genes (Table S4), but we have studied developing rather than differentiated tissue.

Overall, our results are consistent with a role for chromatin structure in the tissue-specific regulation of gene expression during development [18].

### The Epidermal Differentiation Complex (EDC)

The most strikingly significant physical cluster was pC-KD4 from chromosome 1p21. pC-KD4 arises within the EDC, a locus known to hold families of genes expressed during terminal epidermal differentiation [19], [20]. It covers a region of approximately 2.5 Mb on chromosome 1p21 [21]. EDC proteins are primarily localized within or beneath the cornified envelope [22].

Members of the EDC that were differentially expressed in our experiment included *FLG* (C3), *NICE-1* (C3), *SPRL1B* (C3), *SPRR3* (exC3), *SPRR2B* (exC3), *LOR* (C3), *S100A9* (exC3), *S100A8* (exC3), *SPRR1B* (exC5), *PGLYRPIbeta* (C5), *THH* (exC6), *IVL* (exC6), *SPRR1A* (exC6), *S100A12* (exC6), *S100A7* (exC6), *S100A10* (exC8) and *LEP7* (exC11). *SPRR* and *LEP* genes encode precursor proteins of the cornified cell envelope [23], [24]. FLG, IVL and THH are thought to provide physical toughness to the epidermis. The S100A calcium-binding proteins are secreted during inflammation and have a wide range of immunological and antimicrobial actions [25], [26], [27], [28], [29].

Most pC-KD4 genes showed increasing levels of abundance during differentiation and were found in exC3 and exC6. *S100A10* by exception declined in transcript abundance (exC8). An additional *S100* gene, *S100P* was co-expressed in exC3 from chromosome 4p16. The presence of so many EDC genes in exC3 further supported the importance of this cluster to epithelial barrier and defence formation (Table 2).

### The Major Histocompatability Complex (MHC)

**Figure 5:**
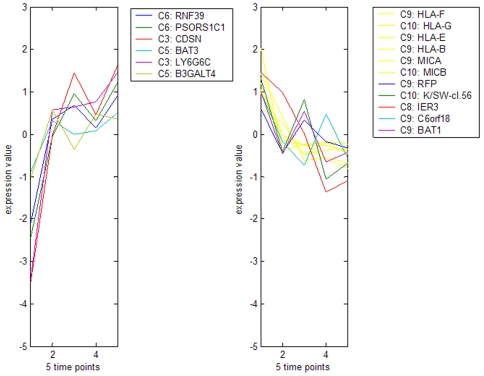
Expression of MHC genes during keratinocyte differentiation. The figure shows abundance levels of those transcripts from the extended MHC which vary significantly during keratinocyte differentiation. Gene transcripts of increasing abundance are shown in the left panel, and transcripts of decreasing abundance on the right. The expression cluster containing each transcript is listed before each gene symbol.

Another prominent area of physical clustering was found within the extended MHC on chromosome 6p21.3 (Figure 5). The region contained pC-KD12, which held the genes for *CDSN* (exC3), *BAT3* (exC5), *PSORS1C1* (exC6), *IER3* (exC8), C6orf18 (exC9), *HLA-B* (exC9), *MICA* (exC9), *BAT1* (exC9), beta 5-tubulin (*OK/SW-cl.56* in exC10) and *MICB* (exC10). The function of many of these genes is not known. The presence of genes showing increasing and decreasing transcript levels (Figure 5) in the same pC indicates a complex regulation of expression within this region.

Other differentially expressed transcripts from the MHC included *HLA-F* (exC9), *HLA-G* (exC10), and *HLA-E* (exC9). The expression of these non-classical HLA alleles by keratinocytes has not previously been recognised. Their expression and that of HLA-B declined during differentiation (Figure 5), suggesting that undifferentiated keratinocytes are more active immunologically than differentiated keratinocytes.

An alternative explanation of the decreasing abundance of MHC transcripts during differentiation is that Class I MHC proteins may function in cellular communication during development. This latter role for Class I MHC molecules has previously been observed in developing neurones [30]. It may be relevant that *HLA-G* alleles have been associated in Jewish patients with Pemphigus Vulgaris, an idiopathic blistering disorder characterised by loss of cell-cell adhesion between keratinocytes [31], and that *HLA-G* has been implicated in the aetiology of asthma in Hutterites [32].

### MHC Histone 1 Cluster

A second physical cluster within the extended MHC, pC-KD12, contained specific linker histone 1 genes which were all within exCs of increasing abundance. These were *HIST1H2BG* (exC4), *HIST1H2BE* (exC5), *HIST1H2BC* (exC6), *HIST1H2AC* (exC4) and *HIST1H2BD* (exC6). Two other histone 1 genes, *HIST1H2BK* (exC5) and *HIST1H1C* (exC4), were expressed from the region in non-contiguous DNA. These are all components of a group of approximately 60 histone genes known as *HIST1* within the extended MHC [33].

It has been suggested that this exceptionally large gene cluster may have evolved to deliver the substantial requirement for histone production during the cell cycle [33], [34], but our results suggest a selective rather than global usage of these genes. As the linker histone proteins are polymorphic [34], their selective use may indicate tissue-specific functions of particular histone genes [35].

### Calcium Binding

The differentiation of keratinocytes is triggered by an increase in extracellular calcium ion concentration [36], [37]. A calcium gradient is present in the skin [38] and is lost after barrier perturbation [39]. This provides a potential mechanism for the precise control of keratinocyte division during maintenance and repair of the epidermis.

We therefore identified differentially expressed genes with a GO annotation for calcium ion binding (Table S6). Of these, only *NOTCH2* (exC5) and *NOTCH3* (exC6) also had the ‘regulation of transcription’ term, consistent with the recognised role for NOTCH in keratinocyte development [40]. *JAG1* (exC8), which also binds calcium and encodes the receptor for NOTCH shows progressively decreasing abundance during differentiation. Soluble JAG1 induces keratinocyte differentiation [41], emphasising the importance of this pathway in epidermal development. Other calcium-binding regulators of growth included *EMR2* (exC1), *PPPR2A* (exC4), *ARHT1* (exC4), *FAT* (exC8), *LTBP2* (exC8) and *GAS6* (exC9).

Immune regulators include *CYP27B1* (the Vitamin D receptor) (exC7), *PPP3CA* (exC8) and *PLSCR1* (exC10). PPP3CA (calcineurin A alpha) influences NF-κB and is the target of the cyclosporin immune modulators. These include tacrolimus and pimecrolimus which are potent topical therapies of AD. These drugs have been considered to exert their effect on T-cells, but our findings may suggest an additional keratinocyte-specific action to be exploited therapeutically. Parathyroid hormone-like hormone *PTHLH* (*PTHrP*) is co-regulated with *CYP27B1* in exC7. It does not bind calcium, but instead is a key regulator of calcium metabolism within the cell. PTHLH agonists are effective therapies for psoriasis, an inflammatory skin disease characterised by epithelial hyper-proliferation [42].

Other calcium binding genes exert their actions on cell adhesion. These include the desmogelins *DSG1* (exC3), *DSCG3* (exC5) and *DSG2* (exC10), the desmocollins *DSC1* (exC3) and *DSC3* (exC4)) and other molecules including *NID2* (exC1), *ANAX9* (exC2), *ANAX3* (exC7), *CDH3* (exC8) and *PCDH7* (exC9). Yet other calcium binding genes influence coagulation and haemostasis, including *PROS1* (exC4), *THBD* (exC5), *THBS1* (exC7) and *THBS2* (exC10).

Genes with binding motifs for Ca^++^ may bind other divalent cations, and zinc binding has been shown to confer anti-microbial actions to S100A7 [27] and the S100A8/S100A9 (calgranulin) complex [28]. S100A12 is also antimicrobial [29], potentially through a similar mechanism.

### Disease Associations

The genes identified in this study are involved in numerous disease processes. Psoriasis and AD both show genetic linkage to pC-KD4 on chromosome 1q21 within the EDC [43], and psoriasis shows strong association to pC-KD13 within the MHC [7], [8]. Genes from exC3 which have established roles in AD and psoriasis include *SPINK5* [5], *FLG* [6] and *CDSN* [7], [8]. Other EDC and exC3 members may be strong candidates for genetic mapping studies for both diseases.

PC-KD13 within the MHC contains the psoriasis susceptibility locus, *PSORS1*. Extensive linkage disequilibrium (LD) mapping has limited the locus to three candidates, *CDSN*, *POSRS1C1* and *HLA-C*. The strong LD in the region has made detection of disease-causing alleles difficult [7], [8]. As *HLA-C* was not expressed in differentiating keratinocytes, *CDSN* and *PSORS1C1* may be better candidates than *HLA-C* for this genetic effect.

Chromosomes 3q21, 17q25, and 20p are also linked to AD and psoriasis [43], and differentially regulated genes from these loci (Table S1) may be considered as candidates.

Search of the OMIM databases with differentially regulated genes from our SAM list identified 19 conditions, including 14 single gene disorders affecting the skin (Table S7). Sixteen genes had known associations with malignancy. Six of these were in exC9 and 3 were in exC10, consistent with the excess of genes regulating cell cycle and proliferation identified by our GO analysis. Genes within exC9 in particular may merit systematic investigation for a role in epithelial cancer.

## Discussion

The study of a single human cell type in controlled conditions has shown a high level of organisation of co-ordinate gene expression with physical clustering and sharing of distinctive TF binding sites among co-expressed transcripts. This may present a framework for the investigation of the cell-specific human transcriptome and its regulation. RNAi knockdown of differentially regulated genes and the *in vitro* identification of TF binding sites will allow a systematic investigation of keratinocyte biology similar to that applied to model organisms such as yeast [44].

The epidermal barrier is of fundamental importance to Atopic Dermatitis and many common skin diseases. Our findings provide a systematic description of the anatomy of epidermal barrier formation and will be a reference of wide utility for subsequent understanding of how the barrier functions in health and disease.

Stimulation with pro-inflammatory cytokines or bacterial products may be expected to identify other modules of co-expressed genes. The investigation of other epithelial cell types may be expected to find shared as well as individual clusters of gene expression and genomic localisation that will further define the biology of the human epithelium.

## Methods

### EpiDerm Culture

The EpiDerm™ 200 skin equivalent cultures were prepared at MatTek Corporation following their standard protocol. Normal human epidermal keratinocytes (NHEK) were initially grown on plastic in monolayer culture before harvest by trypsinization. These freshly harvested cells (time 0 days) were then seeded onto collagen-coated microporous membrane inserts (Millicell CM, Millipore Corp., Bedford, MA) in serum-free, low-calcium growth medium and cultured submerged for one day at 37°C in a 5% CO_2_ incubator. The day after seeding, the medium was changed to high calcium to promote differentiation. After two additional days of submerged culture in high calcium medium the inserts were raised to the air-liquid interface by removal of the medium from the apical compartment (time 3 days). Air-liquid interface culture continued for a total of seven days (time 5, 7, 10 days) in order to produce the fully differentiated epidermal equivalents. The culture medium was serum-free throughout the process.

### RNA Isolation

EpiDerm™ cultures were homogenized in lysis buffer and applied to a glass fiber filter spin column for total RNA isolation (RNAqueous®, Ambion, Inc., Austin, TX). Residual DNA contamination was removed by treatment with the DNA-Free™ DNase reagent (Ambion). Total RNA integrity, purity and concentration were evaluated by agarose gel electrophoresis and UV spectrophotometry. RNA purity and quality was further verified by conducting RT-PCR experiments in the presence and absence of reverse transcriptase. Purified total RNA was stored at −70°C until utilized for expression profiling.

### Preparation of Labelled cRNA

Between 5 and 10 micrograms of total RNA was reverse transcribed to double stranded cDNA using a T7-(dT)_24_ primer (ProOligo France SAS, Paris) and SuperScript II reverse transcriptase (Invitrogen, Paisley, UK). cDNA was purified using the GeneChip® Sample Cleanup Module (Affymetrix, Santa Clara, CA, USA). Biotinylated cRNA was synthesised from cDNA using the Enzo BioArray HighYield Transcript Labelling Kit (Enzo Life Sciences, Farmingdale, NY, USA) and purified using the GeneChip Sample Cleanup Module. cRNA quality was assessed on an Agilent 2100 Bioanalyzer (Agilent Technologies, Palo Alto, CA, USA), and quantity determined spectrophotometrically.

### Hybridisation of cRNA to Microarrays

Twenty micrograms of cRNA was fragmented at 94°C for 35 minutes in fragmentation buffer (GeneChip® Sample Cleanup Module) and hybridised onto HG-U133A GeneChip® arrays (Affymetrix) for 16 hours at 45°C. Arrays were washed, stained with biotinylated anti-streptavidin antibody and streptavidin-phycoerythrin and scanned on a GeneArray 2500 scanner (Hewlett Packard).

### Statistical Analysis of Microarray Data

A total of 22,283 probe sets from the HG-U133A were assayed. We used the global method of scaling/normalization (GeneChip® Operating Software; Affymetrix) with a target signal intensity of 100 to allow comparison across all arrays. Raw data files were analysed using the Bioconductor [45] Affymetrix package (http://www.bioconductor.org). Probe set signals were logged base 2 and expression distributions for each replicate were centralised to 0, such that a change in expression of 1 unit would correspond to the usual empirical significance level of a two-fold change in expression. The data was filtered to include only probe sets with robust signal in all 3 replicates at a minimum of one time point. As approximately one third of the probe sets on Affymetrix HG-U133A array detect the same transcript, we consolidated probe sets from the same gene with a Pearson correlation coefficient ≥0.75 into single results. This threshold was determined from the 95% confidence of the distribution of the Pearson coefficient from a random sample of 9982 probe sets: consolidation reduced these to 9136 combined probe sets. Genes who transcript levels were significantly changed during the five differentiation time points were identified by the significance analysis of microarrays (SAM) method ^2^, with an estimated false discovery rate (FDR) <0.01% on the basis of 100 permutations (MEV package).

### K-Means Clustering

Transcripts with closely correlated expression patterns were identified from the SAM list by K-means clustering [46]. For a range of *k* (cluster number), the best outcome based on minimization of within cluster/between cluster Euclidean distance was adopted from 1000 initial random seedings. The optimal number of clusters were estimated using the Silhouette Value (S) [47].

### Gene Ontology

Gene Ontology (GO) annotation of human proteins (GOA-Human, release February 2005; http://www.ebi.ac.uk/GOA/) was mapped onto the probe set of the Affymetrix HG-U133A array using manufacturer supplied protein accession numbers. The frequency of GO terms was compared between all probe sets on the HG-U133A array and probe sets within expression profile clusters. Only GO terms with a depth in the range of 3 to 7 of the ontology (February 2005 monthly release; http://geneontology.org/) were considered. GO terms associated with just a single probe set on the array were excluded from all analyses. Significance was tested with a two-tailed Fishers exact test. We took a conservative approach to correct for multiple testing, using the Dunn-Sidak correction, by correcting for the total number of GO-terms associated with probe sets on the array rather than just the number of terms associated with probe sets in a cluster.

### Transcription Factors

We sought common *cis*-regulatory elements in the upstream regions of transcripts within the same expression cluster 2 Kb upstream and 500 base pairs downstream of a transcription start site (TSS). TSSs were identified as described previously [48]. For each set of sequences we used the Weeder algorithm [49] to scan and identify the 50 highest scoring ‘sequence words’ of lengths 6 and 8 with 1 and 2 mutations respectively. The significance of scores for each word was assessed through 1,000 random samplings of a set comparable set of human promoter sequences. To assess specificity of regulatory elements to each exC, we permuted cluster membership 1,000 times amongst the set of 1,282 statistically significant probesets and repeated the transcription factor annotation.

We identified ‘interesting’ motifs based on the consolidation of similar and nested words into a single motif with degeneracy, and considered the significance of this consolidated motif to be the sum of the significance for all contributing words. The position frequency matrix of consolidated motifs were compared to the matrixes within the TRANSFAC® Release 8.3 database using the T-Reg Comparator[50] analysis tool with a divergence score cutoff of 0.5. In all cases we report the matched matrix with the lowest dissimilarity score. Regulatory themes within exCs were visualised using hierarchical clustering of robust, annotated motifs using binary distance and average linkage.

We identified transcription factors from amongst the differentially regulated transcripts by using the GO Biological Process ‘regulation of transcription’ and Molecular Function ‘transcription cofactor/corepressor activity, transcription activating factor and transcription factor activity’.

### Smith-Waterman (SW) Algorithm and Physical Clustering

We used a modified SW algorithm [15] to search the genome for regions enriched with genes from the SAM list. The normalized D-value from the SAM algorithm was used to represent the extent of differential expression for each probe, logged to the base 2 and normalized into a range from 0 to 1. A constant *(b*) was subtracted from each value in order to make the average below 0, to produce a sequence of values *a_i_*. The S statistic was calculated for each point along the genome according to the equation: *S_i_*  =  max (*S_i−1_ + a_i_, 0*).

The optimum number of significant physical clusters was identified by varying *b* from 0.5 to 0.75 in steps of 0.05, with a final value of *b* = 0.60 chosen for subsequent analyses. The identities of the genes were randomly shuffled along the genome, and the SW algorithm rerun on the resulting spatially-permuted gene expression scores to find the highest-scoring genome-wide segment to occur by chance. This was repeated 10,000 times to obtain the distribution of the highest-scoring genome wide segment score. The actual segments' scores were then compared against this distribution to get their genome-wide significance.

We used the false discovery rate (FDR) to correct for multiple testing, determined from the number of significant islands identified from 10,000 permutations of the data. We chose a FDR <0.033 to define significance as this translated to the false discovery of 1 physical cluster.

## Supporting Information

Figure S1 Keratin genes expressed in Keratinocytes during differentiation
(0.03 MB DOC)

Table S1 Table of gene expression clusters
(0.26 MB XLS)

Table S2 List of transcription factors
(0.25 MB XLS)

Table S3 Gene ontology analysis of expression clusters
(1.12 MB XLS)

Table S4 Physical clusters of genes expressed in Keratinocytes during differentiation
(0.06 MB XLS)

Table S5 Immune genes expressed in Keratinocytes during differentiation
(0.10 MB XLS)

Table S6 Calcium binding genes genes expressed in Keratinocytes during differentiation
(0.02 MB XLS)

Table S7 OMIM list of disease-associated genes expressed in Keratinocytes during differentiation
(0.05 MB XLS)

## References

[1] Liu WM, Mei R, Di X, Ryder TB, Hubbell E (2002). Analysis of high density expression microarrays with signed-rank call algorithms.. Bioinformatics.

[2] Tusher VG, Tibshirani R, Chu G (2001). Significance analysis of microarrays applied to the ionizing radiation response.. Proc Natl Acad Sci U S A.

[3] MacQueen J, Le Cam LM, Neyman J (1967). Some methods for classification and analysis of multivariate observations.. Proceedings of the Fifth Berkeley Symposium on Mathematical Statistics and Probability.

[4] (2001). Creating the gene ontology resource: design and implementation.. Genome Res.

[5] Walley AJ, Chavanas S, Moffatt MF, Esnouf RM, Ubhi B (2001). Gene polymorphism in Netherton and common atopic disease.. Nat Genet.

[6] Palmer CN, Irvine AD, Terron-Kwiatkowski A, Zhao Y, Liao H (2006). Common loss-of-function variants of the epidermal barrier protein filaggrin are a major predisposing factor for atopic dermatitis.. Nat Genet.

[7] Nair RP, Stuart P, Henseler T, Jenisch S, Chia NV (2000). Localization of psoriasis-susceptibility locus PSORS1 to a 60-kb interval telomeric to HLA-C.. Am J Hum Genet.

[8] Veal CD, Capon F, Allen MH, Heath EK, Evans JC (2002). Family-based analysis using a dense single-nucleotide polymorphism-based map defines genetic variation at PSORS1, the major psoriasis-susceptibility locus.. Am J Hum Genet.

[9] Simpson AJ, Maxwell AI, Govan JR, Haslett C, Sallenave JM (1999). Elafin (elastase-specific inhibitor) has anti-microbial activity against gram-positive and gram-negative respiratory pathogens.. FEBS Lett.

[10] Simpson AJ, Wallace WA, Marsden ME, Govan JR, Porteous DJ (2001). Adenoviral augmentation of elafin protects the lung against acute injury mediated by activated neutrophils and bacterial infection.. J Immunol.

[11] Sakata Y, Arima K, Takai T, Sakurai W, Masumoto K (2004). The squamous cell carcinoma antigen 2 inhibits the cysteine proteinase activity of a major mite allergen, Der p 1.. J Biol Chem.

[12] Vasilopoulos Y, Cork MJ, Murphy R, Williams HC, Robinson DA (2004). Genetic association between an AACC insertion in the 3′UTR of the stratum corneum chymotryptic enzyme gene and atopic dermatitis.. J Invest Dermatol.

[13] Reverter D, Maskos K, Tan F, Skidgel RA, Bode W (2004). Crystal structure of human carboxypeptidase M, a membrane-bound enzyme that regulates peptide hormone activity.. J Mol Biol.

[14] Hurst LD, Pal C, Lercher MJ (2004). The evolutionary dynamics of eukaryotic gene order.. Nat Rev Genet.

[15] Price TS, Regan R, Mott R, Hedman A, Honey B (2005). SW-ARRAY: a dynamic programming solution for the identification of copy-number changes in genomic DNA using array comparative genome hybridization data.. Nucleic Acids Res.

[16] Freedberg I, Tomic-Canic M, Komine M, Blumenberg M (2001). Keratins and the keratinocyte activation cycle.. J Invest Dermatol.

[17] Lercher MJ, Urrutia AO, Hurst LD (2002). Clustering of housekeeping genes provides a unified model of gene order in the human genome.. Nat Genet.

[18] Sproul D, Gilbert N, Bickmore WA (2005). The role of chromatin structure in regulating the expression of clustered genes.. Nat Rev Genet.

[19] Mischke D, Korge BP, Marenholz I, Volz A, Ziegler A (1996). Genes encoding structural proteins of epidermal cornification and S100 calcium-binding proteins form a gene complex (“epidermal differentiation complex”) on human chromosome 1q21.. J Invest Dermatol.

[20] Hardas B, Zhao X, Zhang J, Longqing X, Stoll S (1996). Assignment of psoriasin to human chromosomal band 1q21: coordinate overexpression of clustered genes in psoriasis.. J Invest Dermatol.

[21] South A, Cabral A, Ives J, James C, Mirza G (1999). Human epidermal differentiation complex in a single 2.5 Mbp long continuum of overlapping DNA cloned in bacteria integrating physical and transcript maps.. J Invest Dermatol.

[22] Christiano AM (1997). Frontiers in keratodermas: pushing the envelope.. Trends Genet.

[23] Lohman F, Medema J, Gibbs S, Ponec M, van de Putte P (1997). Expression of the SPRR cornification genes is differentially affected by carcinogenic transformation.. Exp Cell Res.

[24] Marshall D, Hardman MJ, Nield KM, Byrne C (2001). Differentially expressed late constituents of the epidermal cornified envelope.. Proc Natl Acad Sci U S A.

[25] Donato R (2001). S100: a multigenic family of calcium-modulated proteins of the EF-hand type with intracellular and extracellular functional roles.. Int J Biochem Cell Biol.

[26] Cookson W (2004). The immunogenetics of asthma and eczema: a new focus on the epithelium.. Nat Rev Immunol.

[27] Glaser R, Harder J, Lange H, Bartels J, Christophers E (2005). Antimicrobial psoriasin (S100A7) protects human skin from Escherichia coli infection.. Nat Immunol.

[28] Clohessy PA, Golden BE (1995). Calprotectin-mediated zinc chelation as a biostatic mechanism in host defence.. Scand J Immunol.

[29] Gottsch JD, Eisinger SW, Liu SH, Scott AL (1999). Calgranulin C has filariacidal and filariastatic activity.. Infect Immun.

[30] Huh GS, Boulanger LM, Du H, Riquelme PA, Brotz TM (2000). Functional requirement for class I MHC in CNS development and plasticity.. Science.

[31] Gazit E, Slomov Y, Goldberg I, Brenner S, Loewenthal R (2004). HLA-G is associated with pemphigus vulgaris in Jewish patients.. Hum Immunol.

[32] Nicolae D, Cox NJ, Lester LA, Schneider D, Tan Z (2005). Fine mapping and positional candidate studies identify HLA-G as an asthma susceptibility gene on chromosome 6p21.. Am J Hum Genet.

[33] Horton R, Wilming L, Rand V, Lovering RC, Bruford EA (2004). Gene map of the extended human MHC.. Nat Rev Genet.

[34] Marzluff WF, Gongidi P, Woods KR, Jin J, Maltais LJ (2002). The human and mouse replication-dependent histone genes.. Genomics.

[35] Khochbin S (2001). Histone H1 diversity: bridging regulatory signals to linker histone function.. Gene.

[36] Hennings H, Holbrook K, Steinert P, Yuspa S (1980). Growth and differentiation of mouse epidermal cells in culture: effects of extracellular calcium.. Curr Probl Dermatol.

[37] Hennings H, Holbrook KA (1983). Calcium regulation of cell-cell contact and differentiation of epidermal cells in culture. An ultrastructural study.. Exp Cell Res.

[38] Elias PM, Nau P, Hanley K, Cullander C, Crumrine D (1998). Formation of the epidermal calcium gradient coincides with key milestones of barrier ontogenesis in the rodent.. J Invest Dermatol.

[39] Mauro T, Bench G, Sidderas-Haddad E, Feingold K, Elias P (1998). Acute barrier perturbation abolishes the Ca2+ and K+ gradients in murine epidermis: quantitative measurement using PIXE.. J Invest Dermatol.

[40] Lefort K, Dotto GP (2004). Notch signaling in the integrated control of keratinocyte growth/differentiation and tumor suppression.. Semin Cancer Biol.

[41] Aho S (2004). Soluble form of Jagged1: unique product of epithelial keratinocytes and a regulator of keratinocyte differentiation.. J Cell Biochem.

[42] Holick MF, Chimeh FN, Ray S (2003). Topical PTH (1-34) is a novel, safe and effective treatment for psoriasis: a randomized self-controlled trial and an open trial.. Br J Dermatol.

[43] Cookson WO, Ubhi B, Lawrence R, Abecasis GR, Walley AJ (2001). Genetic linkage of childhood atopic dermatitis to psoriasis susceptibility loci.. Nat Genet.

[44] Rustici G, Mata J, Kivinen K, Lio P, Penkett CJ (2004). Periodic gene expression program of the fission yeast cell cycle.. Nat Genet.

[45] Gautier L, Cope L, Bolstad BM, Irizarry RA (2004). affy–analysis of Affymetrix GeneChip data at the probe level.. Bioinformatics.

[46] Tavazoie S, Hughes JD, Campbell MJ, Cho RJ, Church GM (1999). Systematic determination of genetic network architecture.. Nat Genet.

[47] Rousseeuw PJ (1987). Silhouettes: a graphical aid to the interpretation and validation of cluster analysis.. J Comp App Math.

[48] Carninci P, Kasukawa T, Katayama S, Gough J, Frith MC (2005). The transcriptional landscape of the mammalian genome.. Science.

[49] Pavesi G, Mauri G, Pesole G (2001). An algorithm for finding signals of unknown length in DNA sequences.. Bioinformatics.

[50] Roepcke S, Grossmann S, Rahmann S, Vingron M (2005). T-Reg Comparator: an analysis tool for the comparison of position weight matrices.. Nucleic Acids Res.

