# Supplementary material for: Dynamic and Physical Clustering of Gene Expression during Epidermal Barrier Formation in Differentiating Keratinocytes
Source: PLoS One. 2009 Oct 30;4(10):e7651. doi: 10.1371/journal.pone.0007651 (PMC2766255; doi:10.1371/journal.pone.0007651)
Supplement: Supplementary file 1 [file pone.0007651.s001.doc]

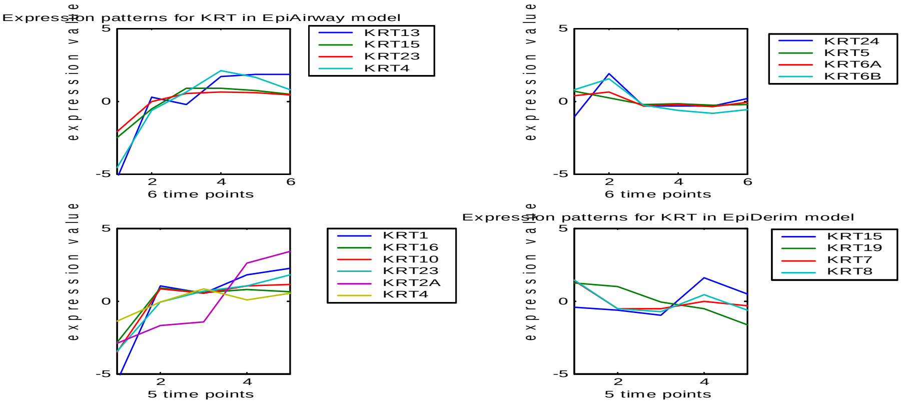
Expression patterns for KRT in EpiAirway model
expression value
6 time points
KRT13
KRT15
KRT23
KRT4
KRT24
KRT5
KRT6A
KRT6B
Expression patterns for KRT in EpiDerim model
5 time points
KRT1
KRT16
KRT10
KRT23
KRT2A
KRT4
KRT15
KRT19
KRT7
KRT8
